# Information about Coronavirus Exposure Effects Attitudes Towards Voting Methods

**DOI:** 10.1017/XPS.2020.38

**Published:** 2020-10-09

**Authors:** Alauna C. Safarpour, Michael J. Hanmer

**Affiliations:** 1 Department of Government and Politics, University of Maryland, College Park, MD, 20742, USA; 2 Center for Democracy and Civic Engagement, University of Maryland, College Park, MD, 20742, USA

**Keywords:** voting methods, in-person Election Day voting, in-person early voting, voting by mail, survey experiment

## Abstract

The COVID-19 pandemic dramatically altered all aspects of life, including the creation of trade-offs between the right to vote and health. While many states postponed primary elections, Wisconsin forged ahead with their April 7, 2020 primaries. The result was widely criticized, with health officials raising concerns about the spread of COVID-19 through in-person voting. We argue that concerns from Wisconsin health officials about the potential to contract COVID-19 via in-person voting can shift American’s comfort with using various voting methods in November. We test our hypotheses using a survey experiment on a diverse national sample. We find that information about possible coronavirus exposures decreases comfort with voting in-person yet does not increase comfort with voting by mail. We discuss the implications, including the need to tailor messages to specific features of various methods of voting in order to increase citizens’ comfort with voting in upcoming elections.

Election administration and individual political behavior were not immune to the massive global disruption of COVID-19. U.S. institutions and citizens faced early tests when primary elections were to occur when medical and social science offered limited guidance.

Unlike many states, Wisconsin’s attempts to postpone their elections during a state stay-at-home order were thwarted by the legislature. Additionally, the U.S. Supreme Court ruled ballots had to be postmarked by Election Day, limiting absentee options.^1^ The election was subsequently described as a debacle,^2^ fiasco,^3^ and disaster.^4^ Long lines and crowded polling places prompted health officials to express concerns with viral spread.

Using a survey experiment on a diverse national sample, we examine the effect of information from Wisconsin’s Health Services about possible COVID-19 infections due to in-person voting on comfort with various voting methods. Results indicate information about coronavirus exposures following Wisconsin’s election decreases comfort voting in-person early and on Election Day yet does not significantly increase comfort with mail voting.

## Expectations

Psychologists agree that individuals are motivated, at least in part, by self-preservation (Muraven and Baumeister 1997). It is reasonable that individuals will feel anxiety at the prospect of contracting a deadly disease and that their self-preservation instinct will guide behavior under such circumstances. Indeed, a national survey weeks before Wisconsin’s election found large majorities worried about contracting coronavirus and took steps to avoid illness including by maintaining physical distance from others and staying home as much as possible.^5^ In this context, we expect individuals to view news of infections linked to in-person voting through the lens of self-preservation and for this to impact comfort with voting methods. Individuals may also be guided by concern over infecting others, and thus altruism may impact comfort with voting in-person.

Political science has long attended to how structural aspects of the electoral process shape attitudes and behavior. For example, Alvarez, Hall, and Llewellyn (2008) show confidence one’s vote was accurately counted varies by method. During the pandemic, self-interest or concern about infecting others may reasonably play a larger role, particularly for in-person voting which brings interactions with others. Given the increased health risks we propose:

H1: Comfort with in-person voting will decrease when individuals learn about potential COVID-19 spread during in-person voting.

Given attempts to postpone elections and increase absentee voting, it is reasonable that most believed mail voting did not pose health risks. But in-person voting worries might not translate into greater comfort with a method less familiar to most. Outside health, mail voting might raise concerns with fraud, undue influence, privacy, and potential for missing late-breaking news (Gronke et al. 2008). Additionally, while support for mail voting is high in states that have it (Southwell 2004), Alvarez et al. (2011) show low support for entirely mail elections. Absentee voting has expanded greatly since Alvarez et al.’s study, suggesting overall support has grown as well. Hassell (2017) finds that voters can be persuaded to choose mail over in-person voting, suggesting preferences on voting methods are malleable. Overall, whether comfort with mail voting is influenced by information about health risks is less clear, but we test the following:

H2: Comfort with mail voting will increase when individuals learn about potential COVID-19 spread during in-person voting.

## Design

Between April 28 and 30, 2020, Qualtrics recruited a diverse sample of 1,313 adult citizens for an online survey and randomly assigned them to either a control or treatment condition.^6^ All participants were first told “As you may know, Wisconsin recently held primary elections with in-person voting.” Treatment participants then read an excerpt from a recent Wisconsin Department of Health Services’ post:

“Today the Department of Health Services (DHS) announced new tracing mechanisms for local health departments to better track Wisconsin residents who may have been exposed to COVID-19 during Tuesday’s election.”

Participants then rated their comfort voting in-person on Election Day, during early voting, and voting by mail in the November election (in that order). Because comfort is linked to self-reported likelihood of voting (see online Appendix), comfort with particular vote methods are meaningful outcomes of interest.^7^

## Results

**Figure 1:**
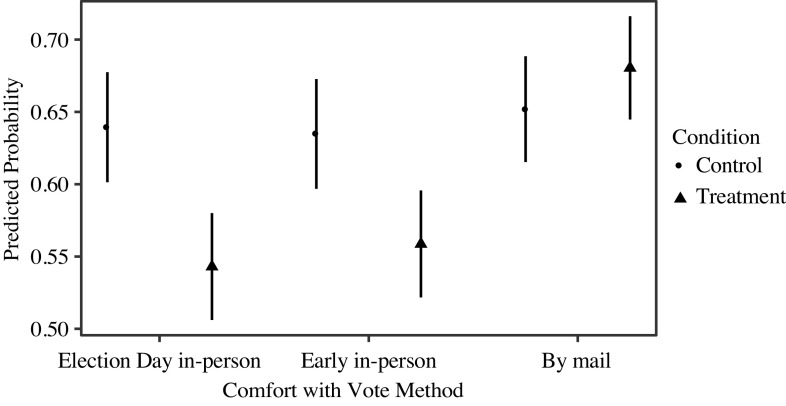
Treatment Effect on Comfort with Vote Methods. *NOTES*: Estimates calculated using OLS regression. Error bars are 95% confidence intervals. Respondents asked: “In November, how comfortable or uncomfortable would you be [voting in person at a polling place on Election Day/voting in person at an early voting location before Election Day/voting with a ballot you receive and return by mail]?” Dependent variables coded 1 for very/somewhat comfortable, 0 for neither comfortable nor uncomfortable and somewhat/very uncomfortable.

The probability of expressing comfort with each voting method by condition is displayed in Figure 1. For each method, we code very/somewhat comfortable as 1, and 0, otherwise, and use Ordinary Least Squares (OLS) regression. Since the dependent and independent variables are binary, this is a more efficient difference-of-means test (Hanmer and Kalkan 2013).

Subjects shown information about possible coronavirus exposure while voting expressed significantly less comfort voting in-person. The treatment reduced the probability of expressing comfort with in-person, Election Day voting by about 10% points (*p* < 0.001, two-tailed), and reduced comfort with early voting in-person by about 8% points (*p* = 0.005, two-tailed). Given the treatment did not provide evidence of actual infections, but just suggested the possibility, these are fairly large effects. The treatment did not significantly increase comfort with mail voting (*p* = 0.27, two-tailed). While the treatment did not directly target a shift to mail voting, structural differences between in-person and mail voting suggest future research might explore focused messaging to increase comfort with mail voting.

## Conclusion

Running elections during COVID-19 requires balancing concerns over providing access and ensuring safety. An original experiment demonstrates that voting-linked coronavirus infections news reduces comfort voting in-person. Interestingly, comfort with mail voting, the main alternative, does not increase in tandem.

Other COVID-19 research suggests the need for tailored messaging to alter some attitudes and behavior (Kuipers, Mujani, and Pepinsky 2020). That literature along with our results has implications for election administration during health crises. Given continuing media coverage of coronavirus infections, election officials should expect to tailor messages to specific features of voting methods to increase citizens’ comfort. This will likely involve convincing voters opting to vote in-person that they will be safe and alleviating concerns with mail voting.

## Footnotes

1
*Republican National Committee v. Democratic National Committee*, No. 19A1016 (U.S. Apr. 6, 2020).

2
https://time.com/5818773/wisconsin-coronavirus-elections/.

3
https://www.politico.com/news/agenda/2020/04/21/simplest-way-avoid-wisconsin-fiasco-election-day-196625

4
https://www.chicagotribune.com/politics/elections/ct-nw-nyt-wisconsin-election-problems-20200410-rdea6424ynecjemkwwfyjqcyqq-story.html

5
https://www.washingtonpost.com/politics/poll-finds-universal-lifestyle-changes-rising-stress-and-growing-fears-about-catching-coronavirus/2020/03/26/11360bb2-6f5e-11ea-b148-e4ce3fbd85b5_story.html

6 Additional details in the online Appendix.

7 Given the time between Wisconsin’s primary and November, we did not expect privacy concerns to play a role. Additionally, results from Horvath, Banducci, and James (2020) suggest reduced emphasis on privacy during the pandemic.

## Supplementary material

For supplementary material accompanying this paper visit https://doi.org/10.1017/XPS.2020.38. click here to view supplementary material

## References

[ref1] Alvarez, R. Michael , Thad E. Hall , Ines Levin , and Charles Stewart III . 2011 Voter Opinions About Election Reform: Do They Support Making Voting More Convenient? Election Law Journal 10(2): 73–87.

[ref2] Alvarez, R. Michael , Thad E. Hall , and Morgan H. Llewellyn . 2008 Are Americans Confident Their Ballots Are Counted? The Journal of Politics 70(3): 754–66.

[ref3] Gronke, Paul , Eva Galanes-Rosenbaum , Peter A. Miller , and Daniel Toffey . 2008 Convenience Voting. Annual Review of Political Science 11: 437–55.

[ref4] Hanmer, Michael J. and Kerem Ozan Kalkan . 2013 Behind the Curve: Clarifying the Best Approach to Calculating Predicted Probabilities and Marginal Effects from Limited Dependent Variable Models. American Journal of Political Science 57(1): 263–77.

[ref10] Hassell, Hans J.G. 2017 Teaching Voters New Tricks: The Effect of Partisan Absentee Vote-By-Mail Get-Out-the-Vote Efforts. Research and Politics (January–March): 1–6.

[ref5] Horvath, Laszlo , Susan Banducci , and Oliver James . 2020 Citizens’ Attitudes to Contact Tracing Apps. Journal of Experimental Political Science 2020: 1–27. doi: 10.1017/XPS.2020.30.

[ref6] Kuipers, Nicholas , Saiful Mujani , and Thomas Pepinsky . 2020 Encouraging Indonesians to Pray From Home During the COVID-19 Pandemic. Journal of Experimental Political Science 2020: 1–12. doi: 10.1017/XPS.2020.26.

[ref7] Muraven, Mark and Roy F. Baumeister . 1997 Suicide, Sex, Terror, Paralysis, and Other Pitfalls of Reductionist Self-Preservation Theory. Psychological Inquiry 8(1): 36–40.

[ref8] Safarpour, Alauna C. and Michael J. Hanmer . 2020 Replication Data for: Information about Coronavirus Exposure Effects Attitudes Towards Voting Methods. Harvard Dataverse. doi: 10.7910/DVN/5E1PNE.

[ref9] Southwell, Priscilla L. 2004 Five Years Later: A Re-assessment of Oregon’s Vote By Mail Electoral Process. PS: Political Science & Politics 37(1): 89–93.

