# Supplementary material for: Information about Coronavirus Exposure Effects Attitudes Towards Voting Methods
Source: Journal of Experimental Political Science. 2020 Oct 9:1–5. doi: 10.1017/XPS.2020.38 (PMC7683816; doi:10.1017/XPS.2020.38)
Supplement: Supplementary file 1 [file S205226302000038Xsup001.docx]

# **Supplementary Materials for “Information about Coronavirus Exposure Effects Attitudes Towards Voting Methods” by Alauna C. Safarpour and Michael J. Hanmer**

## **APPENDIX A: Survey Information and Materials Referenced in Manuscript**

### *Subjects and Context:*

Subjects were eligible to participate if they were over the age of 18, a U.S. citizen, and consented to participate. The survey firm Qualtrics was used to recruit participants. Qualtrics recruited participants across a range of different panel partners. Participants were emailed an invitation to complete a brief survey on current events. All participants completed the survey between April 28-30, 2020. More information on Qualtrics’ procedures is available at: <https://www.qualtrics.com/research-services/online-sample/>

In total, 1,313 individuals consented to participate in the survey, 639 were randomly assigned to the control and 674 were randomly assigned to the treatment. In the control, one individual broke off the survey and did not complete any of the post-treatment survey questions bringing the total analyzed in the control to 638. In the treatment condition, one individual skipped the early voting and mail voting questions bringing the total analyzed in the treatment condition to 673 for comfort voting early and by mail, and 674 for comfort voting in-person on election day. Because attrition was so low once individuals consented to participate, analysis of attrition by demographic indicators is not possible. See CONSORT flow diagram in Appendix C for additional details.

No weighting procedures were used prior to the analysis.

**Table A1. Sample Demographics.**

| Gender | 52% Female  48% Male |
| --- | --- |
| Age | Mean=45 Range: 18-89 |
| Party | 24% Strong Democrat  14% Not very strong democrat  7% Lean Democrat  13% Independent (no leaners)  7% Lean Republican  11% Not very strong Republican  24% Strong Republican |
| Education | 0.15% 8^th^ grade or less  3% Some High School  20% HS Graduate  21% Some college  13% Associate’s degree  25% Bachelor’s Degree  18% Graduate Degree |
| Ideology | 10% Very Liberal  9% Liberal  9% Leans Liberal  32% Moderate  9% Leans Conservative  13% Conservative  17% Very Conservative |

### *Randomization Check:*

Randomization was performed at the individual level using random assignment features in the survey software Qualtrics. Tests indicate successful randomization as condition assignment was not a function of age, sex, party, ideology, and education: $\chi^{2}= 4.30, p=0.64.$

### *Relationship between Comfort and Likelihood of Voting:*

An August 2020 national probability sample from *The Washington Post-University of Maryland* survey, conducted by Ipsos using the KnowledgePanel, shows that comfort is strongly related to the probability of voting and probability of voting in-person. The following tables show the respective cross-tabulations.

More information concerning the sample, methodology, and survey questionnaire is available at: <https://www.washingtonpost.com/context/washington-post-university-of-maryland-poll-by-ipsos-on-voting-issues-aug-24-31/d0ca1dc3-4b85-40b1-92b1-4c91986d0be3/?itid=lk_inline_manual_2>

**Table A2: Relationship Between Likelihood of Voting and Comfort Voting In-Person**

|  |  | **Absolutely Certain to Vote** | **Probably Will Vote** | **Chances are 50-50** | **Less than 50-50 Chance** | **Total** |
| --- | --- | --- | --- | --- | --- | --- |
| **Very comfortable** | N | 467 | 23 | 6 | 24 | 521 |
|  | Percent | 90 | 4 | 1 | 5 | 100 |
| **Somewhat comfortable** | N | 347 | 46 | 30 | 50 | 474 |
|  | Percent | 73 | 10 | 6 | 11 | 100 |
| **Somewhat uncomfortable** | N | 399 | 42 | 32 | 55 | 528 |
|  | Percent | 76 | 8 | 6 | 10 | 100 |
| **Very uncomfortable** | N | 287 | 20 | 26 | 49 | 381 |
|  | Percent | 75 | 5 | 7 | 13 | 100 |
| **Total** |  | 1501 | 131 | 94 | 179 | 1905 |
| Source: *The Washington Post-University of Maryland* poll conducted August 24-31, 2020 by Ipsos. | | | | | | |
|  |  |  |  |  |  |  |

**Table A3: Relationship Between Likely Voting Method and Comfort Voting With Various Methods**

|  |  | **In-Person** | **By Mail** | **Drop Off** | **Total** |
| --- | --- | --- | --- | --- | --- |
| **Very comfortable** | N | 423 | 52 | 47 | 521 |
|  | Percent | 81 | 10 | 9 | 100 |
| **Somewhat comfortable** | N | 285 | 117 | 70 | 474 |
|  | Percent | 60 | 25 | 15 | 100 |
| **Somewhat uncomfortable** | N | 161 | 238 | 126 | 528 |
|  | Percent | 31 | 45 | 24 | 100 |
| **Very uncomfortable** | N | 83 | 228 | 67 | 381 |
|  | Percent | 22 | 60 | 18 | 100 |
| **Total** |  | 952 | 634 | 310 | 1905 |
| Source: *The Washington Post-University of Maryland* poll conducted August 24-31, 2020 by Ipsos. | | | | | |

### *Regression Results:*

Table A4 displays the regression results used to construct Figure 1 in the main text of the manuscript.

**Table A4. Treatment Effect on Comfort with Vote Methods (Binary Outcomes).**

|  | | | |
| --- | --- | --- | --- |
|  | Dependent Variable: Comfort with Vote Method | | |
|  |  | | |
|  | In Person on Election Day | Early in Person | By Mail |
|  | (1) | (2) | (3) |
|  | | | |
| Treatment | -0.096^***^ | -0.076^***^ | 0.028 |
|  | (0.027) | (0.027) | (0.026) |
|  |  |  |  |
| Constant | 0.639^***^ | 0.635^***^ | 0.652^***^ |
|  | (0.019) | (0.019) | (0.019) |
|  |  |  |  |
|  | | | |
| Observations | 1,312 | 1,311 | 1,311 |
| R^2^ | 0.010 | 0.006 | 0.001 |
| Adjusted R^2^ | 0.009 | 0.005 | 0.0001 |
| Residual Std. Error | 0.490 (df = 1310) | 0.490 (df = 1309) | 0.472 (df = 1309) |
| F Statistic | 12.712^***^ (df = 1; 1310) | 7.912^***^ (df = 1; 1309) | 1.196 (df = 1; 1309) |
|  | | | |
| Notes: ^***^p<0.01. OLS estimates. Standard errors in parentheses. Respondents asked: “In November, how comfortable or uncomfortable would you be [voting in person at a polling place on Election Day/ voting in person at an early voting location before Election Day/ voting with a ballot you receive and return by mail]?” Dependent variables are coded 1 for very/somewhat comfortable with vote method, 0 for neither comfortable nor uncomfortable and somewhat/very uncomfortable. | | | |

The following regression table (A5) and Figure A1 demonstrate that the substantive findings reported in the main text which utilize a binary recoding of the dependent variables and OLS regression hold when analyzed using the original response options and ordered logistic regression.

**Table A5. Treatment Effect on Comfort with Vote Methods (Ordered Outcomes).**

|  | | | |
| --- | --- | --- | --- |
|  | Dependent Variable: Comfort with Vote Method | | |
|  |  | | |
|  | In Person on Election Day | Early in Person | By Mail |
|  | (1) | (2) | (3) |
|  | | | |
| Treatment | 0.403^***^ | 0.385^***^ | -0.193^*^ |
|  | (0.100) | (0.099) | (0.101) |
|  |  |  |  |
| Very comfortable\| Somewhat comfortable | -0.456^***^ | -0.560^***^ | -0.293^***^ |
|  | (0.076) | (0.077) | (0.076) |
|  |  |  |  |
| Somewhat comfortable\| Neither | 0.574^***^ | 0.589^***^ | 0.596^***^ |
|  | (0.077) | (0.077) | (0.078) |
|  |  |  |  |
| Neither\| Somewhat uncomfortable | 1.455^***^ | 1.599^***^ | 1.532^***^ |
|  | (0.086) | (0.088) | (0.090) |
|  |  |  |  |
| Somewhat uncomfortable\| Very uncomfortable | 2.697^***^ | 2.739^***^ | 2.275^***^ |
|  | (0.118) | (0.120) | (0.110) |
|  |  |  |  |
|  | | | |
| Observations | 1,312 | 1,311 | 1,311 |
|  | | | |
| Notes: ^*^p<0.1; ^**^p<0.05; ^***^p<0.01. Ordered logit estimates. Standard errors in parentheses. Respondents asked: “In November, how comfortable or uncomfortable would you be [voting in person at a polling place on Election Day/ voting in person at an early voting location before Election Day/ voting with a ballot you receive and return by mail]?” | | | |

**Figure A1. Treatment Effect on Comfort with Vote Methods (Ordered Logit).**

The following regressions shown in Table A6 and Table A7 demonstrate that our findings hold even after accounting for vote method used in 2016.

**Table A6. Comfort with Voting in Person on Election Day Robustness Checks.**

|  | | | |
| --- | --- | --- | --- |
|  | Dependent Variable: Comfort with Voting in Person on Election Day | | |
|  |  | | |
|  |  | | |
|  | (1) | (2) | (3) |
|  | | | |
| Treatment | -0.096^***^ | -0.085^***^ | -0.108^***^ |
|  | (0.027) | (0.026) | (0.026) |
|  |  |  |  |
| Voted in Person on Election Day in 2016 |  | 0.314^***^ |  |
|  |  | (0.026) |  |
|  |  |  |  |
| Independent |  |  | -0.174^***^ |
|  |  |  | (0.041) |
|  |  |  |  |
| Republican |  |  | 0.184^***^ |
|  |  |  | (0.028) |
|  |  |  |  |
| Constant | 0.639^***^ | 0.472^***^ | 0.592^***^ |
|  | (0.019) | (0.023) | (0.024) |
|  | | | |
| Observations | 1,312 | 1,312 | 1,311 |
| R^2^ | 0.010 | 0.111 | 0.072 |
| Adjusted R^2^ | 0.009 | 0.110 | 0.070 |
| Residual Std. Error | 0.490 (df = 1310) | 0.464 (df = 1309) | 0.475 (df = 1307) |
| F Statistic | 12.712^***^  (df = 1; 1310) | 81.767^***^  (df = 2; 1309) | 33.684^***^  (df = 3; 1307) |
|  | | | |

Notes: ^***^p<0.01. OLS estimates. Standard errors in parentheses. Respondents asked: “In November, how comfortable or uncomfortable would you be voting in person at a polling place on Election Day?” Dependent variables are coded 1 for very/somewhat comfortable with voting in person on Election Day, 0 for neither comfortable nor uncomfortable and somewhat/very uncomfortable. Voted in-Person on Election Day in 2016 is coded as 1 if individual voted in person on election day in 2016, 0 is coded as voting early in person, voting by mail, or did not vote in 2016. Excluded party in model 3 is Democrats.

**Table A7. Comfort with Voting Early in Person Robustness Checks.**

|  | | | |
| --- | --- | --- | --- |
|  | Dependent Variable: Comfort with Voting Early in Person | | |
|  |  | | |
|  |  | | |
|  | (1) | (2) | (3) |
|  | | | |
| Treatment | -0.076^***^ | -0.079^***^ | -0.085^***^ |
|  | (0.027) | (0.027) | (0.026) |
|  |  |  |  |
| Voted Early in Person in 2016 |  | 0.116^***^ |  |
|  |  | (0.043) |  |
|  |  |  |  |
| Independent |  |  | -0.224^***^ |
|  |  |  | (0.042) |
|  |  |  |  |
| Republican |  |  | 0.126^***^ |
|  |  |  | (0.028) |
|  |  |  |  |
| Constant | 0.635^***^ | 0.623^***^ | 0.615^***^ |
|  | (0.019) | (0.020) | (0.024) |
|  |  |  |  |
|  | | | |
| Observations | 1,311 | 1,311 | 1,310 |
| R^2^ | 0.006 | 0.012 | 0.058 |
| Adjusted R^2^ | 0.005 | 0.010 | 0.055 |
| Residual Std. Error | 0.490 (df = 1309) | 0.488 (df = 1308) | 0.477 (df = 1306) |
| F Statistic | 7.912^***^ (df = 1; 1309) | 7.631^***^ (df = 2; 1308) | 26.605^***^ (df = 3; 1306) |
|  | | | |
| Notes: ^***^p<0.01. OLS estimates. Standard errors in parentheses. Respondents asked: “In November, how comfortable or uncomfortable would you be voting in person at an early voting location before Election Day?” Dependent variables are coded 1 for very/somewhat comfortable with voting early in person, 0 for neither comfortable nor uncomfortable and somewhat/very uncomfortable. Voted Early in Person in 2016 is coded as 1 if individual voted early in person in 2016, 0 is coded as voting in person on election day, voting by mail, or did not vote in 2016. Excluded party in model 3 is Democrats. | | | |

### **APPENDIX B: Experimental Manipulation and Question Wording**

This study was part of a longer survey. Only questions used in our analysis are included here.

### *Experimental Manipulation:*

Control Condition:

As you may know, Wisconsin recently held primary elections with in-person voting.

When you are ready, please proceed to the next page to answer some questions about voting.

Treatment Condition:

As you may know, Wisconsin recently held primary elections with in-person voting. Please read the following excerpt from a recent post by the Wisconsin Department of Health Services:

 “Today the Department of Health Services (DHS) announced new tracing mechanisms for local health departments to better track Wisconsin residents who may have been exposed to COVID-19 during Tuesday’s election."

When you are ready, please proceed to the next page to answer some questions about voting.

*Note:* While we did not include a manipulation check survey question, respondents who sped through the survey (categorized as less than one-third the median completion time established in a soft launch of our study) were disqualified.

### *Dependent Variables Question Wording:*

1. In November, how comfortable or uncomfortable would you be voting in person at a polling place on Election Day?
2. In November, how comfortable or uncomfortable would you be voting in person at an early voting location before Election Day?
3. In November, how comfortable or uncomfortable would you be voting with a ballot you receive and return by mail?

Response options for all DVs:

-Very comfortable

-Somewhat comfortable

-Neither comfortable nor uncomfortable

-Somewhat uncomfortable

-Very uncomfortable

Order of DVs was not randomized.

### *Demographic and Covariate Questions:*

Age: How old are you? [Dropdown menu to select an age between 18 and 96 or older)

Sex: Please select your sex. [Male/ Female/Other]

*Party:*

Generally speaking, do you usually think of yourself as a ...?

-Democrat

-Republican

-Independent

-Other

[If Democrat or Republican] Would you call yourself a strong [Democrat/Republican] or a not very strong [Democrat/Republican]?

-Strong [Democrat/Republican]

-Not very strong [Democrat/Republican]

[If Independent or Other] Do you think of yourself as closer to the...?

-Democratic Party

-Republican Party

-Neither

*Ideology:* We hear a lot of talk these days about liberals and conservatives. On a 7-point scale, where 1 is very liberal and 7 is very conservative, where would you place yourself on this scale, or haven’t you thought much about this?

-1 Very Liberal

-2

-3

-4 Moderate

-5

-6

-7 Very Conservative

-8 Haven’t thought much about this

*Education:* What is the highest level of school you have completed or the highest degree you have achieved?

-8th grade or less

-Some high school

-High school graduate - high school diploma or equivalent (for example: GED)

-Some college but no degree

-Associate's degree in college

-Bachelor's degree in college (for example: BA, AB, BS)

-Graduate degree (for example: MA, MS, MBA, MD, JD, PhD)

*Vote_2016:* Did you happen to vote in the last presidential election, when Donald Trump ran against Hillary Clinton, Gary Johnson and Jill Stein, or did you skip that one?

-Yes

-No

-I was not eligible

*VoteMethod_2016:* [Asked if Vote_2016=Yes] Did you vote...

-In person on Election Day (at a polling place or precinct)

-In person before Election Day

-By mail or absentee ballot by mail

### **APPENDIX C: CONSORT Flow Diagram for: “Information about Coronavirus Exposure Effects Attitudes Towards Voting Methods” (2020)**

## Follow-Up

Analysed (n=638)

## Analysis

Analysed: (674 for in-person Election Day, 673 for early and by mail)
♦ Excluded from analysis (break-off) (n= 1)

Lost to follow-up (skipped question) (n= 1)

Discontinued intervention (break-off skipped Early Voting and Mail Voting Questions) (n= 1)

Lost to follow-up (skipped question) (n= 1)

## Enrollment

Allocated to Control (n=639)

♦ Received allocated intervention (n=638)

♦ Did not receive allocated intervention (break-off) (n= 1)

## Allocation

Allocated to Treatment (n=674)

♦ Received allocated intervention (n= 674)

Randomized (n=1,313)

Excluded (n=1052)

♦  Declined to participate (n=109)

♦  Break-off’s after consent, speeders, or Spam (n=943)

Assessed for eligibility (n=2365) )
